# How does the Chinese digital transformation of enterprises affect the auditor switch?

**DOI:** 10.1371/journal.pone.0302013

**Published:** 2024-09-17

**Authors:** Ruohao Liang

**Affiliations:** 1 School of Economics and Management Northwest University, Xi’an, China; Sunway University, MALAYSIA

## Abstract

The digital transformation of enterprises has brought great changes to the audit service demand of enterprises and the audit service supply of auditors. Therefore, there is a pressing need to study the digital transformation of enterprises and its impact on auditor switch. This paper aims to explore the influence of enterprise digital transformation on auditor switch and its potential implications for improving auditor efficiency. Using Python’s machine learning tools and text analysis methods, This paper measure the digital transformation of Chinese listed companies and study the impact of digital transformation on the frequency of auditors witches. Our findings suggest that companies that have undergone digital transformation have reduced the frequency of changing auditors by alleviating information asymmetry, enhancing the effectiveness of companies’ internal controls and increasing audit fees. This unique measurement mode of digital transformation provides new evidence for the relationship between the audit service demand of enterprises and the audit service supply of auditors under the digital transformation environment. The research can assist businesses in understanding how digital transformation affects auditing professionally and in improving their audit processes accordingly.

## 1 Introduction

In the fiercely competitive landscape of the information economy, the digital business model emerges as a novel trajectory [1], prompting Chinese companies to accelerate their digital transformation efforts. Typically, the digital business model undergoes a progression from establishing digital capabilities to digital consumption, culminating in full-scale digital transformation [2]. The transformation of an enterprise into a fully digital entity is a gradual process that cannot be achieved overnight. It often takes years, if not decades, for a listed company of considerable size to complete this journey [3]. Throughout this transformational period, enterprises typically navigate through the strategic planning stage of digital transformation, followed by independent research and development or outsourcing to acquire digital technologies, and finally, the application stage where digital outcomes are implemented [4]. In different stages of digital transformation, enterprises face different internal and external risks. These risks may lay great hidden trouble to the internal management, external evaluation and even existing operations of enterprises. The auditor’s switch is the external reflection of these enterprises’ current operations and expected huge hidden dangers in the future. Because when the business operation is blocked or the prospect is uncertain, it is extremely easy to cause auditor switch. Therefore, our research objectives is to deeply understand how enterprises should face the related risks brought by digital transformation while enjoying the benefits of digital transformation by studying the frequency of changing auditors in the process of digital transformation, so as to provide management reference for other enterprises that are about to or are undergoing digital transformation, and provide practical reference for auditors when facing digital transformation customers.

I carry out this study for a number of several other reasons. First, the development of the global digital economy and the application of digital technology in enterprises have made great changes in the audit paradigm. However, by analyzing the current status of enterprise digitization research and the related documents of auditor switches and digital auditing, it is found that no scholars have conducted in-depth research on the direct relationship between enterprise digitization transformation and auditor switches. Second, The audit demand of enterprises will change with the information asymmetry, the effectiveness of internal control and the increase of audit costs. Whether the audit service supply of auditors can match the audit service needs of enterprises in digital transformation has become a decisive factor in determining the change of auditors in digital enterprises. However, there is no research on the influence of the digital transformation enterprises on the auditor reform from both the audit needs of the enterprises in digital transformation and the supply of digital audit services of auditors, and the influence of the information transparency, the effectiveness of internal control and the change of audit fees on the auditor switch in the process of digital transformation of enterprises. However, at present, the impact of enterprise digital transformation on auditor change has not been studied from two aspects: the audit demand of enterprise digital transformation and the supply of auditor digital audit service, as well as the perspective of information transparency, internal control effectiveness and audit fee change in the process of enterprise digital transformation. Third, From the perspective of research methods, no scholars have adopted python’s machine learning tools and the text analysis method of annual reports of listed companies to carry out empirical research based on multiple regression on the degree of corporate digital transformation and auditor switch. In order to answer the above questions, This paper uses python language machine learning tool and text analysis method of annual reports of listed companies to measure the degree of digital transformation of Chinese listed companies and the strategic planning stage of digital transformation of enterprises, the digital technology research and development learning stage and the digital results application stage, and carries out multiple regression analysis with auditor switches and other control variables.

The possible contribution of this paper as follows: firstly, the existing literatures on digital transformation of enterprises have not yet involved the relationship between the digital transformation and the auditor switch, and there have been many disputes on the positive and negative effects of the digital transformation on enterprises. Through empirical tests, this paper finds that the enterprises in the digital transformation will reduce the frequency of the auditor switch, which proves that the digital transformation brings obvious positive effects to the enterprises, so the digital transformation is an important strategic choice for enterprises in the current economic environment; Secondly, the existing enterprise digital transformation literatures usually regards the digital transformation as a whole process, and has not yet been refined into the enterprise digital transformation strategic planning stage, digital technology R&D learning stage and digital results application stage. This paper enriches the empirical research on enterprise digital strategy deployment, digital technology transformation and digital results application and auditor switch through text analysis. Lastly, the current research on the auditor switch mainly focuses on the traditional characteristics of the enterprise. From the micro perspectives of information asymmetry, internal control quality and audit cost, this paper deeply studies the relationship and mechanism between the digital transformation of the enterprise and the auditor switch. The results are helpful to provide references for auditors to make decisions in the face of increasing business related to digital transformation of customer enterprises. the rest of this study is organized as follows. Section 2 describes the Literature and hypotheses regarding the relationship between digital transformation of enterprises and auditor switch. Section 3 explains our empirical strategy to identify the relationship between digital transformation of enterprises and auditor switch. Section 4 describes the data used in our empirical analysis. Section 5 further explores the mediating mechanism of the relationship between digital transformation of enterprises and auditor switch. The final section provides concluding remarks.

## 2 Literature and hypotheses

### 2.1 Literature review

#### 2.1.1 Digital transformation of enterprises

The digitization of the global economy represents a natural progression driven by the dynamic advancement of information and communication technology. Currently, the level of digital maturity varies significantly among Central and Eastern European countries, with many lagging behind their EU counterparts [5]. In Russia, digital business transformation stands as a top priority across all industries [6], while in Croatia, the focus lies on organizing small and medium-sized enterprises and digitizing human resources [7]. Similarly, Ukraine is actively devising innovative investment management mechanisms to drive business digitization [8]. In today’s evolving landscape, embracing digital transformation is crucial for organizational success. Some research highlights its numerous benefits and necessities. Such as digital transformation influences strategic development, organizational culture, structure, and leadership [9]. Enterprises must adopt new business models and overhaul operations to stay competitive [10, 11]. Various digital technologies, like IoT, enterprise social networking, and big data analytics, support smarter internet products and services [12]. These technologies enhance connectivity, efficiency, and innovation, aiding enterprise digital transformation [13]. At the same time, existing studies have found that digital transformation can enable enterprises to enhance operational capabilities [14], mitigate risks [15], improve innovation performance [16] and improve production efficiency [17, 18].

#### 2.1.2 Influence of enterprise digitalization on audit

The digital transformation of enterprises has also promoted the application of digital audit technology, digital audit methods and tools by auditors in the audit process, and further developed digital audit theory. On the one hand, digital technology makes auditors more efficient, on the other hand, the new digital audit business brings challenges to auditors in unknown areas. While auditors enjoy the improvement of work efficiency brought by digital technology, they are also facing an unprecedented increase in the difficulty of digital audit business. Such as Albitar et al. (2021) stress its consequences on audit costs [19], human capital, procedures, and quality. Nicoleta et al. (2023) highlight auditors’ increased responsibilities and risks with remote audits [20]. Digital skills enable auditors to enhance fraud risk assessments using tools like distributed systems [21]. However, challenges arise in obtaining reliable audit evidence and ensuring data security [22].

Specifically, the digitization of enterprises has transformed audit practices, introducing new methods and tools. The digitization of the economy has reshaped traditional audit practices, moving towards intelligent audits. This shift is fueled by the necessity to adapt to digital environments, fundamentally changing auditing’s cognitive processes. Digital audit is recognized as a distinct scientific field [23]. It offers superior outcomes by exploring new business areas, improving data analysis, developing innovative audit models, fostering innovation, and limiting managerial discretion [24]. It enhances human-computer interaction, improving audit quality [25]. Gronsund et al. (2020) observed a reconfiguration of auditors and algorithms due to organizational algorithm analysis [26]. Auditors cannot be replaced by algorithms alone but need to expand into new areas. Tiron-Tudor et al. (2021) emphasize the interplay between algorithms and human intelligence [27], stressing the importance of human-computer interconnection in enhancing audit quality and verification effectiveness. Digital audit methods cover various aspects such as evidence investigation, financial report analysis, and data storage in digital environments. Such as tools like the digital evidence bag (DEB) acquire and process digital evidence from diverse sources [28]. Real-time digital tech helps secure financial analysis programs [29]. In addition, a lightweight digital evidence preservation system ensures privacy, anonymity, and operational efficiency. A significant advancement is the use of signature public key technology in audits. It dynamically controls tasks and grants permissions during audits, enhancing authenticity and integrity [30]. It also proves the sequence and logic of audit procedures and verifies maliciously modified records [31]. This technology revolutionizes digital audits, providing efficiency and security benefits.

### 2.2 Theoretical framework

From the perspective of the audit service needs of digital transformation enterprises, the audit is mainly responsible for combing and verifying the company’s operating performance and expected risks. Company’s owners and relevant stakeholders need to obtain reliable company operation evaluation through external audit services, so as to carry out relevant business strategy decision-making, investment and financing activities. In the process of choosing audit services, companies will also consider auditor characteristics such as audit fees, expertise, reputation and scale, and tend to choose auditors that best match the characteristics of the enterprise itself.

From the point of view of the audit service supply of auditors, the development strategy, industry attributes and operating risks of client companies are also the focus of auditors’ attention, and auditors pay special attention to the degree of information asymmetry and the effectiveness of internal control in enterprises. Because the degree of information asymmetry increases the risk of audit errors, which would lead to the damage of auditors’ reputation and even the risk of being punished by regulatory authorities; The effectiveness of corporate internal control is closely related to the audit conclusion issued by the auditor. The low effectiveness of internal control may lead to the audit failure or audit corruption faced by the auditor.

In the process of enterprise digital transformation, the audit service needs of enterprises will switch with the asymmetry of information, the effectiveness environment of internal control and the increase of audit costs. Whether the audit service supply of auditors can match the audit service needs of enterprises in digital transformation has become a decisive factor to determine the switch of auditors in digital enterprises. First, severe information asymmetry within an enterprise usually leads to auditor changes, because it would increase the risk of audit errors. When the risk of audit errors is reduced, auditors will actively maintain the audit contract relationship with the company due to the reputation mechanism; Secondly, when the auditor detects that the company has excessive operating risks and internal control risks, it may issue a modified audit opinion, which would damage the audit contract relationship between the enterprise and the auditor; Lastly, there is a non-negligible connection between auditors’ professional knowledge and individuals, which can improve performance in the audit process, and then affect audit costs and audit changes. Therefore, audit costs are also an important factor that determines auditors’ switches. Based on the above relationship between supply and demand, this paper puts forward the theoretical relationship between enterprises in digital transformation and their auditor changes.

### 2.3 Research hypothesis

As can be seen from the above literature review, On the one hand, the digital transformation has caused drastic changes in the internal and external environment of the enterprise, which may affect the audit needs of the enterprise; On the other hand, the digital transformation of enterprises has promoted the transformation from traditional auditing to digital auditing. From the digital audit technology, the application of digital audit methods and tools, and the digital audit theory, there have been brand-new developments. Therefore, with the development of enterprise digital transformation, subversive changes will occur in the audit service demand of enterprises and the audit service supply of auditors in the digital environment, and these changes will be reflected in the auditor changes in time. Resource dependence theory (RDT) posits that organizations depend on external resources to survive and thrive, and their behavior is shaped by their need to acquire and manage these resources effectively. In the context of auditor-client relationships, this theory suggests that companies rely on auditors not only for financial oversight but also for valuable resources such as expertise, credibility, and assurance, which are essential for maintaining stakeholder trust and accessing capital markets. First, As companies undergo digital transformation, they often invest in advanced technologies, data analytics tools [32], and automated systems to improve efficiency, decision-making, and risk management. By enhancing their internal control environments and financial reporting processes through digitalization, companies may reduce their reliance on auditors for certain tasks previously performed manually. For instance, automation can streamline data gathering and processing, making financial information more accurate and readily available [33]. Consequently, auditors may perceive lower risks associated with the audit engagement, potentially decreasing the likelihood of auditor switches.

second, Digital transformation can facilitate greater transparency and real-time access to financial and operational data for both companies and auditors. Through interconnected systems and digital platforms, auditors can gain deeper insights into clients’ operations, transactions [34], and risk profiles [35]. Enhanced visibility into the client’s business processes reduces information asymmetry between auditors and clients, enabling more effective audits and reducing the need for auditor switches driven by concerns about undisclosed risks or misstatements.

Third, RDT emphasizes the importance of trust and interdependence in organizational relationships. Digital transformation can foster closer collaboration and communication between companies and auditors, strengthening their mutual dependence. For example, auditors may provide advisory services related to cybersecurity [36], data integrity [37], or regulatory compliance in the digital realm [38], deepening their engagement with clients beyond traditional audit functions. By demonstrating expertise in navigating digital complexities and adding value beyond compliance, auditors can cultivate stronger relationships with clients, thereby reducing the propensity for auditor switches.

last, As companies embrace digitalization, their strategic priorities and risk profiles may evolve. Auditors need to adapt their audit approach and expertise to address emerging risks and challenges in the digital landscape effectively. Firms that demonstrate a strong alignment between their digital strategies and audit needs are more likely to retain their auditors over the long term [39]. Effective communication and collaboration between companies and auditors regarding digital initiatives and risk mitigation strategies can foster trust and minimize the likelihood of auditor switches driven by perceived misalignment or dissatisfaction. In summary, digital transformation has the potential to reduce the frequency of auditor switches by reshaping the dynamics of auditor-client relationships through improved access to technological resources, reduced information asymmetry, strengthened trust and relationship building, and enhanced strategic alignment. By leveraging digital capabilities to enhance transparency, efficiency, and collaboration, companies can cultivate more stable and mutually beneficial partnerships with auditors, thereby mitigating the need for frequent auditor changes. Therefore, based on the above analysis, the baseline hypothesis H1 is proposed as follows:

**H1: The higher the degree of company digital transformation, the lower the frequency of auditor switch.**

Digital transformation can ease information asymmetry and reduce the risk of audit errors. Enterprises will widely adopt digital technologies such as "big data, artificial intelligence, mobile technology, cloud technology" in the digital transformation. Among them, big data can provide deep mining, secure storage and precise analysis of data; Artificial intelligence can be connected with the Internet of Things technology to realize automatic information collection and non-text information transformation and processing; The widespread use of mobile technology and cloud servers provides reliable technical support for the whole process monitoring of enterprise production and operation [40]. In addition, the characteristics of irreversible modification of blockchain technology have greatly enhanced the reliability and timeliness of enterprise production and operation information [41]. At this time, the business processes of enterprises are becoming more transparent, and the problem of information asymmetry is effectively alleviated. At this point, the financial data used by auditors to perform audits are accurate, reliable, and timely, and the risk of audit errors by the audit team will be reduced. Based on the above, this paper come to hypothesis H2:

**H2: Enterprises alleviate information asymmetry through digital transformation, thus reducing the frequency of auditor switch;**

Digital transformation can enhance the effectiveness of enterprises’ internal controls and reduce operational risks. The digital transformation of enterprises is a process of reshaping business models and optimizing business structures through digital strategic planning, digital technology updating and the application of digital achievements. Therefore, in the digital transformation, the organizational structure of the enterprise tends to be flattened [42], the organizational management efficiency is optimized, and the control level of the organizational structure is enhanced. Previous studies have shown that the internal personnel management, risk control and cost calculation of the enterprise will also realize the integration of the flow of financial authority and the flow of affairs authority [43]. In addition, the digital decision-making system will effectively inhibit the irrational factors of management decision-making and rent-seeking behavior, thereby improving management efficiency and quality, and reducing the company’s operating risks. At the same time, Chung (2019) analyzes data from 4,568 company years on EJCho’s Korean stock market, revealing unions’ impact on external auditor tenure [44]. Heliodoro (2016) identifies a positive correlation between qualified audit reports and auditor switches, especially concerning assets [45]. Hassan et al. (2018) highlight board effectiveness as a determinant of auditor switches [46], emphasizing governance’s role. These studies collectively emphasize audit risk’s role in auditor changes, considering factors like EQCR times, labor dynamics, audit opinions, and governance structures. Based on the above analysis, this paper further puts forward hypothesis H3:

**H3: Enterprises improve the effectiveness of internal control through digital transformation, thus reducing the frequency of auditor switch;**

Digital transformation will lead to higher audit fees. The digital transformation of enterprises challenges the traditional audit methods and tools in accounting & audit practice, and urges auditors to continuously invest in digital related resource endowments to develop new audit tools and methods that conform to the development of digital technology and digital economy. In this environment, many auditors have actively responded to the impact of the digital economy and carried out digital audit technology and strategic transformation. For example, Deloitte’s financial robot, Ernst & Young’s global audit digital platform "EYCanvas", PwC artificial intelligence system "GL.ai", KPMG’s artificial intelligence tool "Ignite", and the digital cloud platform of Chinese registered auditors "CICPA.COM" were constructed and used. Auditors have made additional investment in digital transformation in response to the audit business of digital transformation of enterprises, so they will also charge higher audit fees than traditional audit services for enterprises with digital transformation services. The complexity of audit operations significantly increases audit fees, and as the digital transformation of the client company progresses, the audit team will inevitably face incremental digital audit operations, thus increasing audit fees. At this time, auditors are more likely to actively maintain audit contractual relationships with the client company for the sake of profitability and reducing the additional transaction costs of finding new customers. So this paper put forward hypothesis H4:

**H4: Due to the digital transformation, enterprises increase audit fees, thus reducing the frequency of auditor switch.**

## 3 Research design

### 3.1 Sample selection and data source

This paper takes Chinese listed companies as research samples, and the research range is from year 2007 to 2020. In data processing, this article eliminates samples of companies with abnormal status, companies that have been delisted or suspended from delisting. The company-level data are all from the CSMAR database, and all continuous variables have been winsorised at 1% level. Regression analysis has been performed by stata 15.0 software.

The reason for choosing the range to start from year 2007 is that China Internet companies started the second round of overseas listing boom since 2007, which greatly stimulated the awareness and enthusiasm of Chinese enterprises for digital transformation. This year was called the starting point of digital transformation of enterprises in China. Therefore, this paper takes 2007 as the starting point to study the digital transformation of Chinese enterprises.

### 3.2 Variable definition

#### 3.2.1 Explanatory variable: Digital transformation of enterprises

At present, the indicators of digital transformation of enterprises are mainly described by text analysis of annual reports. Based on the text analysis and word frequency statistics of the annual report of listed companies, this paper takes the word frequency of digital transformation keywords in the annual report as a proxy index to measure the level of digital transformation of enterprises. The specific steps are as follows:

1. Build a text library. With the crawler technology of Python language, a total of 43,413 annual financial reports of 4,896 China A-share listed companies from 2007 to 2020 were collected on the website of Juchao Information, and the "From PDF Miner. PDF Interpimport PDFresourceManager, PDFPageInterpreter, Third-party libraries in Python languages, such as PDFTextExtractionNotAllowed, convert the PDF version of the annual financial report of listed companies into TXT format as the text library of enterprise digital transformation characteristic words.
2. Determine the characteristic lexicon of enterprise digital transformation. Based on the definition and statistical methods of enterprise digital transformation in the existing literature, the characteristic vocabulary of enterprise digital transformation is defined from three dimensions: strategic planning of enterprise digital transformation, learning stage of digital technology research and development and application stage of digital achievements, and the characteristic vocabulary of enterprise digital transformation is formed as shown in Table 1.
3. Calculate the word frequency of enterprise digital transformation characteristic words. Based on the text database generated in the early stage and the characteristic words in Table 1, the machine learning command in Python language is used to search, match and count the characteristic words in Table 1, and the total index of digital transformation is obtained. In the process of word frequency statistics using Python language, firstly, "import jieba.analyse" means reading the document and calling jieba to divide words, and combining with UTF-8, Chinese words are recognized. Secondly, specific Python language command packages such as "from PDF miner. Layout Import LTTextboxHorizontal, La Params" are used to identify, capture and count the text content in PDF format, and a stoplist is also needed. Finally, the number of occurrences of specific keywords is counted and matched with other related variables. The detailed index is generated by the word frequency of three dimensions: the strategic planning of enterprise digital transformation, the research and development stage of digital technology and the application stage of digital achievements.

**Table 1:** Digital transformation characteristic word library of enterprises.

	Dimension division	Characteristic words
Digital transformation	Digital transformation strategy is planned by stages.	Internet Times, Internet thinking, Internet business model, digitalization, intelligence, financial technology, platformization, converged architecture, business intelligence, ecosystem, data center, data resources, digital industry, digital innovation, digital transformation, digital management, digital capability, digital transformation, digital currency, digital technology, digital finance, digital economy, digital platform, digital age, digital marketing.
	Digitized achievement Application stage	E-commerce, P2P, online, industrial internet, interconnection, internet, internet finance, internet medical care, open banking, quantitative finance, identity verification, investment decision support system, network connection, unmanned retail, virtual reality, mobile internet, mobile payment, billion-level concurrency, heterogeneous data, augmented reality, credit reporting, smart agriculture, smart wear, smart big data, smart grid, smart environmental protection.
	Digital technology research and development learning stage	Mobile Internet, Internet of Things, artificial intelligence, cloud computing, big data, blockchain, differential privacy technology, third-party payment, multi-party secure computing, distributed computing, machine learning, brain like computing, stream computing, green computing, in-memory computing, embedded analysis, cognitive computing, deep learning, biometric technology, data analysis, data mining, graph computing, image understanding, cyber-physical systems, semantic exploration.

#### 3.2.2 Explained variable: Auditor switch

This paper use whether the enterprise changed the accounting firm in certain year to measure the auditor switch. If the auditor of the listed company switches in the year, the value of switch is 1, otherwise it is 0.

#### 3.2.3 Control variables

Drawing on the relevant research of previous scholars, the control variables are selected as the variables which may influence the relationship between the digital transformation of enterprises and the change of auditors. Specifically, This paper selected three types of control variables: company characteristic variables, company financial characteristic variables, and auditor characteristic variables.

The company characteristic variable is measured by the increase rate of owners’ equity. The growth rate of owner’s equity represents the actual income that the company’s shareholders can obtain from the company’s operation and development, and is a market-oriented performance of the company’s property rights characteristics. Variables of the company’s financial characteristics include debt ratio, degree of excessive liabilities, accounts receivable scale, and growth rate of total operating costs. The asset-liability ratio measures the characteristics of an enterprise’s asset and liability structure; The degree of excessive debt measures the part of corporate debt that exceeds the reasonable scale of debt, and represents the degree of corporate excess debt risk; On the one hand, the scale of accounts receivable represents the potential return risks of the enterprise, on the other hand, it represents the characteristics of the interest relationship between the enterprise and the upstream and downstream industry chains; The growth rate of total operating costs not only represents the cost control level of enterprises, but also represents the speed of business expansion of enterprises.

The auditor characteristic variable is measured by the auditor signing delay. The number of days between the balance sheet date and the signing date of the audit report by the auditor is referred to as the audit time lag. The longer the auditor’s signature delay is, the auditor has greater doubts about the audit results of the business, and it is likely that non-standard audit opinions will be presented. At this time, the more likely the enterprise is to purchase the auditor’s audit opinions. The auditor signing lag therefore represents the degree of difficulty that auditors face in the face of corporate digital transformation and other daily business. In addition, the article also controls the corporate individual effect and the time effect. All variables are shown in **Table 2**.

**Table 2:** Introduction to table variables.

Variable definition	Variable name	symbol	Variable description
Explained variable	Auditor switch	Switch	Whether the company changed its accounting firm in that year
Explanatory variable	Digital transformation	DIG	Number of total word frequencies related to digital transformation in annual reports of listed companies
	Digital technology research and development learning stage	TECH	Number of words frequency about digital technology application in annual reports of listed companies
	Digitized achievement Application stage	ACHIEVE	Number of words used in the annual reports of listed companies about digital achievements
	Digital transformation strategy is planned by stages	STRATEGY	Number of words about digital strategic planning in annual reports of listed companies
Control variable	Asset-liability ratio	TDR	Total ending liabilities divided by total ending assets
	Total operating cost growth rate	COST	(Total operating cost amount in the current period of this year-total operating cost amount in the same period of last year)/total operating cost amount in the same period of last year
	Owner’s equity growth rate	EQUITY	(end value of owner’s equity in the current period-end value of owner’s equity in the same period last year)/end value of owner’s equity in the same period last year
	Accounts receivable	AR	Ending book amount of enterprise accounts receivable
	Auditor’s signature lag	SIGN	Time interval between the end date of the previous fiscal year and the date when the auditor signed the audit report in
	Excessive debt degree	DEBT	Actual debt ratio-target debt ratio

### 3.3 Model design

In order to study the impact of digital transformation of enterprises on the stability of audit contracts, and then test hypothesis 1, I establishes an empirical model (1) as follows:

$${\mathrm{Switch}}_{i,\;t}=\beta_{0}+\beta_{1}{\mathrm{DIG}}_{i,\;t}+\Sigma {\mathrm{Control}}_{i,\;t}^{’}\theta +\Sigma {\mathrm{ind}}_{j}+\Sigma {\mathrm{Year}}_{t}+\varepsilon_{i,\;t}$$ (1)

In the main regression model, i represents the enterprise and t represents the time. Controls i,t refers to all control variables of enterprise i in year t. The specific variable of audit contract stability of the explanatory variable is whether to change the auditors (Switch_i,t_), the explanatory variable is the degree of digital transformation of the enterprise(DIG_i,t_), and Control is the control variable vector; ε is a random error term; At the same time, this paper also controls the Year fixed effect and industry fixed effect.

Furthermore, This paper use the method and procedure of Wen (2015) for reference to test the mediating effect [47]. The specific test equations are shown in models (2)-(7).

According to the three-step method required by mediating effect, the specific research steps are as follows: The first step is to use the model (1) to test whether the impact ***β***_***1***_ of the digital transformation of enterprises on the auditor switch is significantly negative. Secondly, models (2), (4) and (6) are used to test whether the influence coefficient ***α***_***1***_ of enterprise digital transformation on intermediary variables "information asymmetry", "enterprise internal control effectiveness" and "audit fees" is significant. In the third step, models (3), (5) and (7) are used to test the influence of digital transformation of enterprises and intermediary variables, such as information asymmetry, internal control effectiveness and audit fees, on the auditor switch. If***δ2*** is significant, it indicates the existence of intermediary effect. Among them, ***δ1*** is not a complete mediating effect, but a partial mediating effect.


$${\mathrm{Meeting}}_{i,\;t}=\alpha_{0}+\alpha_{1}{\mathrm{DIG}}_{i,\;t}+\Sigma {\mathrm{Control}}_{i,\;t}^{’}\psi +\Sigma {\mathrm{ind}}_{j}+\Sigma {\mathrm{Year}}_{t}+\varepsilon_{i,\;t2}$$ (2)

$${\mathrm{Switch}}_{i,\;t}=\delta_{0}+\delta_{1}{\mathrm{DIG}}_{i,\;t}+\delta_{2}{\mathrm{Meeting}}_{i,\;t}+{\mathrm{\Sigma Control}}_{i,\;t}^{’}\xi +{\mathrm{\Sigma ind}}_{j}+{\mathrm{\Sigma Year}}_{t}+\varepsilon_{i,\;t3}$$ (3)

In models (2) and (3), “Meeting” represents the mediating variable "information asymmetry degree". Models (1), (2) and (3) are used to test hypothesis H2, which is he intermediary mechanism of "digital transformation-information asymmetry-auditor switch".


$${\mathrm{Con}}_{i,\;t}=\alpha_{0}+\alpha_{1}{\mathrm{DIG}}_{i,\;t}+{\mathrm{\Sigma Control}}_{i,\;t}^{’}\psi +{\mathrm{\Sigma ind}}_{j}+{\mathrm{\Sigma Year}}_{t}+\varepsilon_{i,\;t2}$$ (4)

$${\mathrm{Switch}}_{i,\;t}=\delta_{0}+\delta_{1}{\mathrm{DIG}}_{i,\;t}+\delta_{2}{\mathrm{Con}}_{i,\;t}+{\mathrm{\Sigma Control}}_{i,\;t}^{’}\xi +{\mathrm{\Sigma ind}}_{j}+{\mathrm{\Sigma Year}}_{t}+\varepsilon_{i,\;t3}$$ (5)

In models (4) and (5), “Con” represents the intermediary variable "effectiveness of internal control". Models (1), (4) and (5) are used to test hypothesis H3, which is the intermediary mechanism of "digital transformation-effectiveness of internal control-auditor switch".


$${\mathrm{Fee}}_{i,\;t}=\alpha_{0}+\alpha_{1}{\mathrm{DIG}}_{i,\;t}+{\mathrm{\Sigma Control}}_{i,\;t}^{’}\psi +{\mathrm{\Sigma ind}}_{j}+{\mathrm{\Sigma Year}}_{t}+\varepsilon_{i,\;t2}$$ (6)

$${\mathrm{Switch}}_{i,\;t}=\delta_{0}+\delta_{1}{\mathrm{DIG}}_{i,\;t}+\delta_{2}{\mathrm{Fee}}_{i,\;t}+{\mathrm{\Sigma Control}}_{i,\;t}^{’}\xi +{\mathrm{\Sigma ind}}_{j}+{\mathrm{\Sigma Year}}_{t}+\varepsilon_{i,\;t3}$$ (7)

In models (6) and (7), Fee represents the intermediary variable "audit fee". Models (1), (4) and (5) are used to test hypothesis H4, which is the intermediary mechanism of "digital transformation-audit fee-auditor switch".

## 4 Empirical analysis

### 4.1 Descriptive statistics

**Table 3** shows the main descriptive statistical results. According to Table 3.

**Table 3:** Descriptive statistics.

*stats*	*N*	*mean*	*sd*	*min*	*max*	*p25*	*p50*	*p75*
*Switch*	23961	0.118	0.322	0	1	0	0	0
*DIG*	23961	0.036	0.082	0	1.571	0.002	0.009	0.031
*TECH*	23961	0.013	0.043	0	1.388	0	0.002	0.008
*STRATEGY*	23961	0.013	0.038	0	0.842	0	0.002	0.01
*ACHIEVE*	23961	0.010	0.025	0	0.679	0.001	0.003	0.01
*BIG4*	23961	1.938	0.240	1	2	2	2	2
*TOP10*	23961	1.498	0.500	1	2	1	1	2
*STATE*	23961	0.421	0.494	0	1	0	0	1
*TDR*	23961	0.432	0.204	0.007	0.995	0.270	0.428	0.584
*COST*	23961	0.407	13.078	-4.787	1371.69	-0.004	0.119	0.277
*EQUITY*	23961	0.240	4.622	-0.983	631.586	0.018	0.068	0.154
*AR*	23961	1.257	5.897	0.000	180.700	0.110	0.304	0.790
*SIGN*	23961	95.754	20.109	0	838	84	100	112
*DEBT*	23961	-0.006	0.152	-0.607	0.957	-0.107	-0.007	0.095
*Value*	23961	0.020	0.207	0.000	10.710	0.001	0.002	0.008
*IV1-Website*	23961	56.337	9.040	14.7	74	50	57.5	63
*IV2-Internet*	23961	24.399	29.039	0	77.83	0	0	52.9
*Fee*	23961	1.495	3.583	0.1	266.99	0.6	0.895	1.4
*Meeting*	23961	4.815	1.776	0	22	4	4	6
*Con*	23961	6.495	1.209	0	9.954	6.246	6.711	7.045

Data source: Descriptive statistical results of stata15.0

The average frequency of words related to digital transformation in the annual reports of listed companies is 0.036 thousand, with the minimum value of 0 and the maximum value of 1.571 thousand, indicating that the diversity of digital transformation among enterprises is quite large. The average value of the dependent variable is 0.118, which is basically consistent with the existing literatures. The range of other control variables is generally consistent with the existing research.

### 4.2 Results of baseline regression

Column (1) of **Table 4** reports the regression results of the digital transformation of enterprises to the auditor switch. The results show that the influence coefficient of the degree of enterprise digital transformation on Switch is -0.190, which is significantly negatively at the level of 1%.

**Table 4:** Regression results of enterprise digital transformation and auditor switch.

	(1)	(2)	(3)	(4)
*DIG*	-0.190***			
	(-3.54)			
*TECH*		-0.340***		
		(-3.00)		
*STRATEGY*			-0.312***	
			(-2.81)	
*ACHIEVE*				-0.327**
				(-2.16)
*TDR*	0.155***	0.149***	0.149***	0.145***
	(4.67)	(4.52)	(4.50)	(4.39)
*COST*	0.748***	0.750***	0.749***	0.747***
	(3.46)	(3.47)	(3.46)	(3.45)
*EQUITY*	2.151***	2.139***	2.148***	2.143***
	(4.41)	(4.38)	(4.40)	(4.39)
*AR*	0.002**	0.0019**	0.002**	0.0019**
	(2.24)	(2.16)	(2.22)	(2.14)
*SIGN*	0.288**	0.285**	0.284**	0.284**
	(2.35)	(2.32)	(2.32)	(2.31)
*DEBT*	-0.105***	-0.100***	-0.100***	-0.0975***
	(-3.10)	(-2.95)	(-2.95)	(-2.87)
_cons	-0.211	-0.209	-0.208	-0.206
	(-1.64)	(-1.62)	(-1.61)	(-1.59)
Time fixed effect	control	control	control	control
Industry fixed effect	control	control	control	control
*N*	23961	23961	23961	23961
*R* ^2^	0.051	0.051	0.056	0.050

Standard errors in parentheses

* *p* < 0.1

** *p* < 0.05

*** *p* < 0.01

Based on the information provided in Table 1, this study categorizes the digital transformation of enterprises into three dimensions: strategic planning of digital transformation, the stage of digital technology research and development, and the stage of applying digital achievements. The outcomes of this categorization are detailed in columns (2), (3), and (4) of Table 4, and these are further analyzed in relation to whether the enterprise changes its auditor. The results show that the influence coefficients of the strategic planning of enterprise digital transformation, the research and development stage of digital technology and the application stage of digital achievements on auditor switch are -0.340***, -0.312*** and -0.327**, respectively, all of which have significant negative relationships. The results show that digital transformation can significantly reduce the frequency of auditor switches. The empirical results support H1.

The empirical results in column (1) of Table 4 show that the regression coefficient between the asset-liability ratio (TDR) and the auditor switch is 0.155***, indicating that the higher the excessive liabilities, the more likely it is to cause the company’s auditor to switch; The regression coefficient between the owner’s equity growth rate (EQUITY) and the switch of auditors is 2.151***. During the period of rapid growth in revenue, the company will also cause frequent changes in auditors; The regression coefficient between the company’s accounts receivable scale (AR) and the auditor’s switch is 0.002**, which indicates that the higher the company’s income risk and bad debt risk, the easier it is to cause the auditor’s switch; The regression coefficient between the growth rate of total operating costs (COST) and the switch of auditors is 0.748***, indicating that the faster the company’s business expansion, the easier it is for auditors to switch; The regression coefficient between auditor signing delay (SIGN) and auditor switch is 0.288**, which indicates that the auditor is more likely to switch when the auditor faces higher business difficulty, signing doubts and potential audit opinion purchase risk; The degree of excessive liability (DBET) and auditor switch have a regression coefficient of -0.105***, indicating that the company’s asset-liability structure will significantly affect auditor switches.

### 4.3 The intermediary effect test

In the above research hypothesis, I think that the digital transformation can significantly reduce the frequency of the company’s switching auditors for three reasons. First, the process of digitization reduces the asymmetry of internal information, enhances the authenticity of audit data provided to auditors, and reduces the risk of audit errors; Second, the digitization process has enhanced the internal management level and effectiveness of internal controls of enterprises, reduced the company’s own operating risks and the possibility of encountering penalties from regulatory authorities, and helped auditors maintain a good industry reputation; The third is based on the existing research that shows that the digital transformation of enterprises can significantly increase audit fees, so auditors are likely to maintain audit contracts with enterprises due to income factors and transaction costs.

This section will identify and test the above three mechanisms one by one. In view of the fact that the model (1) has been tested in column (1) of Table 4, it is concluded that the regression coefficient of digital transformation on the auditor switch of enterprises is significantly negative. Therefore, the following identification test idea in this section is to adopt models (2)-(7). If the corresponding variables all pass the significance test, it indicates that the corresponding intermediary effect is established.

#### 4.3.1 Information asymmetry

This paper use "Audit Committee Meetings" to measure the degree of information asymmetry encountered by the audit team during the audit work. The more audit committees are held, the more severe the fog of information within the company, and the lower the transparency of the information. The results of the identification test for the mediation mechanism of "Digital Transformation-Information Asymmetry-Auditor Switch" are shown in **Table 5** (1).

**Table 5:** Test results of intermediary effect.

	(1)		(2)		(3)	
	Meeting	*switch*	Con	*switch*	Fee	*switch*
*DIG*	-1.083***	-0.1711***	0.427**	-0.187***	0.672**	-0.1885***
	(-2.99)	(-2.78)	(2.14)	(-2.70)	(2.16)	(-3.47)
Meeting		0.0171***				
		(6.01)				
Con				-0.007***		
				(-3.36)		
Fee					-0.120***	-0.0023**
					(-5.96)	(-2.01)
*TDR*	-0.356	0.196***	-1.668***	0.123***	1.091***	0.152***
	(-1.48)	(3.19)	(-13.68)	(3.43)	(5.56)	(4.50)
*COST*	0.568	3.582***	0.165	0.762***	0.867	0.954***
	(0.13)	(3.23)	(0.23)	(3.66)	(0.69)	(4.39)
*EQUITY*	-5.161	-2.617*	0.524	2.092***	4.100	6.170***
	(-0.94)	(-1.86)	(0.33)	(4.45)	(0.87)	(7.62)
*AR*	0.0068	0.0025**	0.0094***	0.0016	0.0658***	0.0023***
	(1.55)	(2.23)	(2.65)	(1.49)	(12.55)	(2.58)
*SIGN*	-1.176	0.733***	-3.960***	0.381***	1.170	0.279**
	(-1.49)	(3.64)	(-8.88)	(2.91)	(1.62)	(2.25)
*DEBT*	0.111	-0.195***	1.199***	-0.101***	-0.808***	-0.105***
	(0.46)	0.196***	(9.66)	(-2.79)	(-4.03)	(-3.04)
_cons	3.065***	-0.0209	8.471***	-0.0574	0.102	-0.153
	(7.56)	(-0.20)	(64.69)	(-1.36)	(0.13)	(-1.16)
Time fixed effect	control	control	control	control	control	control
Industry fixed effect	control	control	control	control	control	control
*N*	10897	10855	22782	22782	23961	23961
*R* ^2^	0.056	0.054	0.076	0.035	0.056	0.056

Standard errors in parentheses

* p < 0.1

** p < 0.05

*** p < 0.01

The left column of Table 5(1) shows that the digital transformation has a significant negative impact on audit committee meetings (coefficient = -1.083, P < 0.01), indicating that the digital transformation has significantly reduced the number of audit committee meetings, that is, it has effectively alleviated the degree of information asymmetry within the company; Column (1) in Table 5 shows the regression results after the intermediary variables are added, in which the regression coefficient of digital transformation of enterprises (DIG) to auditor switch is -0.1711, which is significantly negatively correlated at the level of 1%;

At the same time, the coefficient of the number of audit committee meetings is 0.0171, which is significantly positively related to the auditor switch at the level of 1%, indicating that the higher the degree of information asymmetry, the easier it is to cause the auditor switch. Combined with the result that digital transformation in the left column of Table 5(1) can significantly reduce the number of audit committee meetings, it shows that the effect of information asymmetry exists. That is, the digital transformation of enterprises significantly reduces the frequency of auditor switch by alleviating the information asymmetry of companies. Hypothesis H2 is verified.

#### 4.3.2 Effectiveness of internal control

This paper uses the DIB Internal Control Effectiveness Index (Con) to measure the effectiveness of the company’s internal controls. The higher the index, the better the company’s internal control, that is, the stronger the operating level and management ability, the lower the operating risk. The results of the identification test of the intermediary mechanism for "Digital Transformation–Effectiveness of Internal Controls–Auditor Switches" are shown in Table 5 (2).

The left column of Table 5(2) shows that digital transformation has a significant positive impact on internal control effectiveness index (coefficient = 0.427, P < 0.1), indicating that digital transformation has significantly enhanced the effectiveness of enterprise internal control; The right column of Table 5 (2) shows the regression results of enterprise digital transformation and internal control effectiveness on auditor switch after adding intermediary variables, in which the regression coefficient of enterprise digital transformation on auditor switch is-0.187, which is significantly negatively correlated at the level of 1%;

At the same time, the regression coefficient of the effective index of internal control (Con) to the auditor switch is -0.007, which is significantly negatively correlated at the level of 1%, indicating that the better the effectiveness of internal control, the lower the frequency of auditor switch. Combined with the results that the digital transformation in the left column of Table 5(2) can significantly enhance the effectiveness of internal control, it shows that some intermediary effects exist in the effectiveness of internal control. That is, digital transformation significantly reduces the frequency of auditor switch by enhancing the effectiveness of internal control. The empirical results support H3.

#### 4.3.3 Audit fees

This paper uses "Fee" to measure audit fees between companies and auditors. The results of the identification test of the mediation mechanism for the Digital Transformation-Audit Fee-Auditor Switch are shown in Table 5 (3). Table 5 (3) The left column shows that the digital transformation significantly affects the audit costs (coefficient = 0.672, p< 0.1), and illustrates the audit costs of enterprises with significant digital transformation, which is consistent with the existing research results [48];

The right column of Table 5 (3) shows the regression results of enterprise digital transformation and audit fees on auditor switchs after the variables are added, in which the regression coefficient of enterprise digital transformation on auditor switchs is -0.1885, which is significantly negatively correlated at the level of 1%;

At the same time, the regression coefficient of audit fees to the auditor switch is -0.0023, which is significantly negatively correlated at the level of 1%, indicating that the higher the audit fees, the lower the switch frequency of auditors in enterprises. Combined with the result that digital transformation in the left column of Table 5 (3) can significantly increase the audit fees, it shows that the intermediary effect of audit fees exists.

With the digital transformation of enterprises, auditors have increased audit fees, and have formed more stable audit contracts with enterprises for profit purposes, thereby significantly reducing the frequency of auditor switches. The empirical results support H4.

To enhance the robustness and statistical power of the mediation effect test in this paper, the Sobel-Goodman Mediation test was employed for a more in-depth analysis of the results presented in Table 5. The outcomes of the Sobel-Goodman Mediation test revealed significantly low P values in columns (2), (4), and (6) of Table 6. This indicates a notable intermediary role played by Information Asymmetry, Effectiveness of internal control, and Audit fees in the context of reducing auditor turnover during the digital transformation of enterprises. Specifically, in the pathway of Information Asymmetry, the direct effect of digital transformation on reducing auditor turnover is -0.1711, with Information Asymmetry contributing an additional intermediary effect of -0.0185 (= 0.0171×-1.083). Regarding the Effectiveness of internal control pathway, the direct effect of digital transformation on reducing auditor turnover is -0.187, and the intermediary role played by Effectiveness of internal control is -0.003 (= 0.427×-0.007). In the Audit fees pathway, the direct effect of digital transformation on reducing auditor turnover is -0.1885, and the intermediary effect of Audit fees is -0.0015 (= 0.672×-0.0023). These results underscore the substantial influence of Information Asymmetry, Effectiveness of internal control, and Audit fees as key factors shaping the dynamics of auditor switches during the digital transformation process in enterprises.

**Table 6:** Sobel-Goodman Mediation test.

	(1)	(2)	(3)	(4)	(5)	(6)
	Information Asymmetry		Effectiveness of internal control		Audit fees	
	Coef	P>|Z|	Coef	P>|Z|	Coef	P>|Z|
Sobel	-0.0185	0.0006	-0.003	0.0002	-0.0015	0.0004
Goodman-1	-0.0185	0.0006	-0.003	0.0002	-0.0015	0.0003
Goodman-2	-0.0185	0.0005	-0.003	0.0001	-0.0015	0.0001
Indirect effect	-0.0185	0.0000	-0.003	0.0000	-0.0015	0.0000
Direct effect	-0.1711	0.0000	-0.187	0.0000	-0.1885	0.0000
Total effect	-0.190	0.0000	-0.190	0.0000	-0.190	0.0000

t statistics in parentheses

* p < 0.1

** p < 0.05

*** p < 0.01

### 4.4 Robustness test

#### 4.4.1 Instrumental variable method

In view of the obvious location agglomeration effect of the digital transformation of enterprises, that is, the development of the overall digital economy in will have a common impact on the digital transformation choices of enterprises in the region. Therefore, this article takes the overall digitization level of the province where the sample company is located as the instrumental variable. Specifically, this paper uses the number of websites per 100 enterprises in the region (IV1-Website) and the regional Internet penetration rate (IV2-Internet) to measure the level of regional digitization. The logic of setting this instrumental variable is that, on the one hand, the geographical digitalization feature is an obvious exogenous variable for the company’s research samples; On the other hand, the number of websites owned by every 100 enterprises and the regional Internet penetration rate are highly related to the degree of digital transformation of sample enterprises in the domain, but they have no direct influence on whether enterprises switch their auditors, so this method meets the setting requirements of tool variables.

Table 7 shows the regression results of instrumental variable method. The left columns of (1), (2), (3) and (4) respectively show the first-stage estimation results of tool variables and enterprise digital transformation, digital technology application, enterprise digital strategic planning and enterprise digital achievement application, and show that the regression coefficients of tool variables, the number of websites owned by every 100 enterprises (IV1-Website) and regional Internet penetration rate (IV2-Internet) on enterprise digital transformation and its three specific dimensional variables are significantly positive, which is in line with the correlation of tool variables.

**Table 7:** Regression results of instrumental variable method.

	(1)		(2)		(3)		(4)	
	*switch*		*switch*		*switch*		*switch*	
*DIG*		-1.930***						
		(-4.18)						
*TECH*				-5.958***				
				(-3.76)				
*STRATEGY*						-4.292***		
						(-4.02)		
*ACHIEVE*								-8.245***
								(-3.96)
*IV1-Website*	0.0006***		0.0002***		0.0003***		0.0001***	
	(7.89)		(4.6)		(8.21)		(4.70)	
*IV2-Internet*	0.0002***		0.0001**		0.0001*		0.0001**	
	(2.79)		(2.40)		(0.146)		(2.38)	
*TDR*	0.0298***	0.165***	0.0104***	0.169***	0.0107***	0.154***	0.0184***	0.178***
	(5.6)	(6.18)	(4.11)	(5.71)	(6.79)	(5.80)	(8.61)	(5.73)
*COST*	0.0528	2.980***	0.0076	2.924***	0.0147	2.940***	0.0003	3.130***
	(0.97)	(2.93)	(0.39)	(2.84)	(1.56)	(3.03)	(1.3)	(2.85)
*EQUITY*	0.1271	4.270***	0.03	4.204***	0.0625	4.293***	0.0002	4.310***
	(1.15)	(2.85)	(0.92)	(2.87)	(1.21)	(2.93)	(0.57)	(2.67)
*AR*	0.0004***	0.0009*	0.0001***	0.0009	0.0001***	0.0007	0.0001**	0.0015
	(2.84)	(1.69)	(3.43)	(1.63)	(4.53)	(1.36)	(2.14)	(1.64)
*SIGN*	0.1412***	0.644***	0.0464***	0.648***	0.0524**	0.595***	0.0001***	0.722***
	(4.4)	(4.36)	(3.12)	(3.93)	(2.26)	(3.91)	(2.82)	(4.51)
*DEBT*	-0.0243***	-0.142***	-0.0104***	-0.157***	-0.0037	-0.112***	-0.019***	-0.179***
	(-3.6)	(-4.45)	(-3.00)	(-4.13)	(-1.14)	(-3.62)	(-7.61)	(-4.49)
*_cons*	-0.0537***	-0.0367	-0.024***	-0.0512**	-0.0134***	-0.0276	-0.01***	-0.0344
	(6.64)	(-1.58)	(-4.06)	(-2.07)	(-4.62)	(-1.14)	(-3.96)	(-1.36)
Time fixed effect	control		control		control		control	
Industry fixed effect	control		control		control		control	
*N*	18280		18280		18280		18280	
*R* ^2^	0.1836		0.1014		0.0971		0.1947	

Standard errors in parentheses

* *p* < 0.1

** *p* < 0.05

*** *p* < 0.01

The right columns of Table 7(1), (2), (3) and (4) are the second stage estimation results. It shows that after the instrumental variables are added, the regression coefficients of digital transformation and its three specific dimensional variables are-1.930、-5.958、-4.292and-8.245 respectively, all of which are significantly negative at the statistical level of 1%. It shows that after controlling endogenous problems by instrumental variable method, the significant reduction effect of digital transformation on the auditor switch still exists.

#### 4.4.2 Other robustness tests

This paper also made the following robustness tests: (1) Propensity Score Matching method (PSM). After matching the enterprises that have implemented digital transformation, This paper re-use the model (1) regression to test the robustness. In the process of matching, all the control variables in model (1) are used as covariates to estimate the tendency score of each enterprise in digital transformation, and the radius matching method is used to complete the matching. The results are listed in Table 8, Column 1.

(2) Advance one period. In order to make the result of regression more robust, the digital transformation of independent variable enterprises will be returned one phase in advance. The results are listed in Table 8, Column 2.
(3) Change the measurement method of variables. While leveraging Python’s machine learning tools and text analysis to gauge the attributes of enterprise digital transformation is both innovative and reliable, it is imperative to acknowledge and address potential sources of bias, data limitations, and the broader applicability of research findings, especially in intricate fields like digital transformation. In light of this, This paper conducted additional measurements of the variables associated with enterprise digitalization transformation, eschewing reliance on machine learning and text analysis. The goal was to assess the stability of the influence of enterprise digitalization on auditor switches.

In this alternative approach, the digitalization level was assessed through the book value of intangible assets linked to digital transformation, as reported in the annual filings of listed companies. To identify these assets, This paper scrutinized intangible asset names for keywords such as software, systems, numbers, information, intelligence, data, communication, media, automation, mobile, network, platform, storage, and others. Projects containing these keywords were categorized as "digital transformation intangible assets," and their values were aggregated to represent an enterprise’s digitalization level for the current year. This method offers an alternative perspective, complementing the machine learning and text analysis approach, and provides a more comprehensive understanding of the impact of digitalization on auditor switches. The results are listed in Table 8, Column 3.

(4) Replace the regression model. In order to make the regression model more robust, a one-dimensional interactive model that controls "time×industry" is adopted for testing. The results are listed in Table 8, Column 4.
(5) Delete the sample of switch reasons of special auditors. In order to control the impact of the auditor switch caused by specific reasons on the conclusion, the samples of auditor switched due to "regular rotation", "merger and renaming" and "switch of management" are eliminated, and the samples of "change of firms without auditor switch" are eliminated and then regression analysis again. The results are listed in Table 8, Column 5.
(6) Delete special year samples. In order to control the external influences of the global financial crisis in 2008, the stock market crash in China in 2015 and the COVID-19 epidemic in 2020 on the digital transformation of listed companies in China, the three-year samples are excluded and regressed. The above robustness test results show that the conclusion is still valid. The results are listed in Table 8, Column 6.

**Table 8:** Regression results of other robustness tests.

	(1)	(2)	(3)	(4)	(5)	(6)
	*switch*	*switch*	*switch*	*switch*	*switch*	*switch*
*F*.*DIG*		-0.134**				
		(-2.11)				
*Value*			-0.0573***			
			(-3.64)			
*DIG*	-0.290***			-0.169***	-0.206***	-0.190***
	(-2.69)			(-3.27)	(-3.39)	(-3.54)
*TDR*	0.186***	0.159***	0.122***	0.110***	0.126***	0.155***
	(3.26)	(4.06)	(2.89)	(3.31)	(3.37)	(4.67)
*COST*	2.467	0.609***	0.735***	0.783***	0.562**	0.748***
	(0.75)	(2.69)	(3.27)	(3.23)	(2.47)	(3.46)
*EQUITY*	-1.535	2.288***	1.980***	1.997***	2.109***	2.151***
	(-0.41)	(4.51)	(3.85)	(3.99)	(4.16)	(4.41)
*AR*	0.0031**	0.0024**	0.0016	0.001	0.0008	0.002**
	(2.29)	(2.26)	(1.36)	(1.05)	(0.71)	(2.24)
*SIGN*	0.223	0.222	0.240	0.422***	0.270*	0.288**
	(1.11)	(1.57)	(1.46)	(3.44)	(1.88)	(2.35)
*DEBT*	-0.189***	-0.100**	-0.0832*	-0.0861**	-0.0675*	-0.105***
	(-3.26)	(-2.54)	(-1.93)	(-2.51)	(-1.75)	(-3.10)
_cons	-0.164	-0.320**	-0.0635	0.0335*	-0.298**	-0.211
	(-1.07)	(-2.13)	(-1.19)	(1.89)	(-2.21)	(-1.64)
Time fixed effect	control	control	control	control	control	control
Industry fixed effect	control	control	control	-	control	control
*N*	23961	21523	18824	22390	22875	21351
*R* ^2^	0.058	0.055	0.0312	-	0.0221	0.051

Standard errors in parentheses

* *p* < 0.1

** *p* < 0.05

*** *p* < 0.01

## 5 Further research

### 5.1 The influence of professional competence of auditors

The digital transformation of enterprises will bring new audit contents including digital assets audit to the audit team. Firms with strong professional competence can provide more professional services, so they can better cope with the challenges of professional knowledge and audit methods brought by digital audit business to audit teams, and thus conclude a more stable audit contract relationship with enterprises. In addition, if the digital transformation leads to the lack of competence of auditors, companies will also voluntarily resign proceeding auditors. This article divides all samples into international "Big Four" firms, Chinese "top ten" firms and other firms, and tests the impact of digital transformation on auditors’ switches in groups. The test results are shown in Table 7. Columns (1) and (2) report the impact of digital transformation on the auditor switch of enterprises when they are audited by international "Big Four" auditors and Chinese "top ten" firms. The regression coefficients are -0.726 and -0.297 respectively, which are significantly negatively correlated at 5% and 1% levels respectively. Table 7, column (3), reports the impact of digital transformation on enterprise switch auditors when audited by other auditors. The regression coefficient is-0.0691, and the regression results are not significant. This shows that in the digital transformation of enterprises, the frequency of switching by auditors with strong professional competence is lower.

### 5.2 Influence of the nature of property rights

The response speed and degree of state-owned enterprises to the national digital development strategy is usually more remarkable, and at the same time, state-owned enterprises are usually subject to more supervision and auditing, and their information transparency and operational risk characteristics are different from those of non-state-owned enterprises. Therefore, the degree, emphasis and strategic direction of digital transformation between state-owned enterprises and non-state-owned enterprises are different, and the impact on the stability of audit contracts may be different. It is necessary to divide all samples into state-owned and non-state-owned ones to test the impact of digitalization on the stability of audit contracts.

This paper divided all sample enterprises into state-owned enterprises and non-state-owned enterprises, and then made regression analysis. The regression results are shown in Table 9. Column (4) reports the influence of digital transformation on the stability of audit relationship contract when the sample is a non-state-owned enterprise. The regression coefficient of the degree of digitalization on the switch of audit is -0.143, which is significantly negatively correlated at the level of 5%. Column (5) reports the impact of digital transformation on auditor switches when the sample is a non-state-owned enterprise. Among them, the degree of digitization has a negative correlation with the regression coefficient of auditors of enterprise switches of-0.272 at the level of 5%. In contrast, state-owned enterprise switch auditors less frequently during the digital transformation process.

**Table 9:** Regression results of heterogeneity test.

	big four	Top ten	others	non-state-owned	state-owned	non-high-tech	high-tech
	*switch*	*switch*	*switch*	*switch*	*switch*	*switch*	*switch*
	(1)	(2)	(3)	(4)	(5)	(6)	(7)
*DIG*	-0.726**	-0.297***	-0.0691	-0.143**	-0.272**	-0.182***	-0.116
	(-2.35)	(-3.42)	(-0.90)	(-2.45)	(-2.00)	(-2.80)	(-0.88)
*TDR*	-0.0598	0.00475	0.196***	0.176***	0.137**	0.141***	0.257*
	(-0.35)	(0.10)	(3.90)	(4.03)	(2.33)	(4.03)	(1.67)
*COST*	0.0037***	0.0032***	0.0006**	0.0008**	0.0009	0.0007***	-0.0280*
	(2.88)	(4.98)	(2.19)	(2.57)	(1.08)	(3.15)	(-1.78)
*EQUITY*	0.0034***	0.0046***	0.0009	0.0018***	0.0099***	0.0021***	0.0336**
	(3.41)	(5.65)	(1.33)	(3.85)	(2.92)	(4.31)	(2.13)
*AR*	0.0024**	0.0027**	-0.0029	-0.006***	0.0031***	0.0019**	0.0050
	(2.08)	(2.54)	(-1.12)	(-2.89)	(2.85)	(2.07)	(0.45)
*SIGN*	0.0004	0.0006***	-0.0001	0.0002*	0.0003	0.0003**	0.0003
	(0.54)	(3.41)	(-0.45)	(1.65)	(1.61)	(2.50)	(0.56)
*DEBT*	0.0018	-0.0075	-0.129**	-0.122***	-0.0844	-0.0946***	-0.125
	(0.01)	(-0.15)	(-2.53)	(-2.80)	(-1.41)	(-2.63)	(-0.91)
_cons	-1.333**	0.260	-0.245	0.145	-0.153	-0.165	0.109
	(-2.56)	(0.82)	(-1.58)	(0.58)	(-0.82)	(-1.16)	(0.23)
Time fixed effect	control	control	control	control	control	control	control
Industry fixed effect	control	control	control	control	control	control	control
*N*	1655	9455	11215	15561	11247	21571	1867
*R* ^2^	0.080	0.056	0.067	0.068	0.049	0.055	0.088

Standard errors in parentheses

* p < 0.1

** p < 0.05

*** p < 0.01

### 5.3 Influence of industry nature

Many high-tech enterprises themselves are the products of the development of digital technology and transformation achievements. Therefore, many digital transformation processes for non-high-tech enterprises may only be routine business areas for high-tech industries. This means that there will be obvious differences in audit strategies, audit emphases and audit methods when auditors face high-tech enterprises and non-high-tech enterprises. There are differences in the choice of auditors between high-tech enterprises and non-high-tech enterprises. Therefore, it is necessary to divide all the samples according to whether they are high-tech industries, and test the impact of digitalization on the auditor switch.

This paper grouped all samples according to whether they are high-tech industries for regression test, and the test results are shown in Table 9. According to the Guidelines for Industry Classification of Listed Companies, information transmission, software and information technology services (I), scientific research and technical services (M) are divided into high-tech industries, while others are classified into non-high-tech industries. Column (6) reports the impact of digital transformation on auditor switches when an enterprise is a non-high-tech enterprise. The regression coefficient of digitization to auditor switches is-0.182, which is significantly negative correlation at the level of 1%. Table 9 Column (7) reports the influence of digital transformation on the stability of audit relationship contract when the enterprise is a high-tech enterprise, and the regression result is not significant. The result shows that compared with high-tech enterprises, the frequency of auditor switch in the process of digital transformation of non-high-tech enterprises is lower.

## 6 Research conclusions

This paper mainly studies the relationship between enterprise digital transformation and auditor switches. This paper find that the frequency of auditor switching can be significantly reduced in every stage of enterprise digital transformation including strategic planning stage, digital technology R & D learning stage and digital results application stage. From the test results of the intermediary mechanism, first of all, the digital transformation of enterprises will reduce the frequency of auditors by reducing corporate information asymmetry, improving the transparency of audit information, and reducing the risk of potential audit errors. Secondly, the process of enterprise digitization optimizes the company’s management efficiency and decision-making level, and enhances the effectiveness of the company’s internal controls, which reduces the probability of auditors issuing non-standard audit opinions, and also reduces the impact of auditors’ purchase of audit opinions, as well as the risk of penalties or reputation damage due to reduced audit quality; Finally, the enterprise digital transformation brings excess business difficulty and working hours to auditors, and also leads to the increase of audit expenses, which in turn promotes the increase of auditors’ compensation. Therefore, for the purpose of profit and avoiding the transaction costs caused by re-finding customers, auditors will also actively maintain audit contract relationships with customer companies, thereby reducing the frequency of auditors’ switches. The four research hypotheses put forward in this paper have been confirmed by empirical research.

From the perspective of practical significance, focusing on the auditor switch is helpful to clarify and analyze the positive effects of digital transformation on enterprises, and has an important practical guiding role for enterprises to correctly select digital transformation models, reasonably avoid digital transformation risks, and effectively improve the quality of digital transformation; From the theoretical point of view, the external audit report provided by auditors can make objective, independent and scientific judgments on the achievements, deficiencies and subsequent development prospects of enterprise digital transformation. If an enterprise has excessive risks in the process of digital transformation, it is likely that the enterprise will adopt the means of purchasing audit opinions to maintain its reputation in the capital market, which will likely lead to the collapse of the contract of audit relationship between the enterprise and the auditor, and also the auditor switch. Research on how the digital transformation of enterprises affects the audit switch can also provide important theoretical reference for the academic circles to understand the current digital transformation of Chinese listed enterprises to achieve results, problems and development directions.

The conclusion of this paper proves that with the development of China’s digital economy and the deepening of global digital strategic competition, the digital transformation of enterprises is not only the historical requirement of the external digital economy environment, but also the inevitable choice for its internal long-term development to obtain endogenous power. Based on the above conclusions, in view of the economic consequences of the digital transformation of enterprises and the follow-up empirical research of enterprise switch auditors, This paper believe that we should pay attention to the follow-up research from the perspective of digital auditing: First, the continuous pursuit of high-quality audits in the digital environment; The second is to attach importance to the adherence to audit ethics and the promotion of audit culture while continuously strengthening digital audit technology.

## Supporting information

S1 Data (XLS)
